# Expedited surgery in geriatric hip fracture patients taking direct oral anticoagulants is not associated with increased short-term complications or mortality rates

**DOI:** 10.1097/OI9.0000000000000089

**Published:** 2020-08-21

**Authors:** Alexander Brown, Zachary Zmich, Aaron Roberts, Jason Lipof, Kyle T. Judd

**Affiliations:** Department of Orthopaedics, University of Rochester School of Medicine and Dentistry, Rochester, New York.

**Keywords:** anticoagulation, direct oral anticoagulation therapy, geriatric hip fracture

## Abstract

**Objectives:**

The purpose of this study was to evaluate potential differences in time to surgery, bleeding risk, wound complications, length of stay, transfusion rate, and 30-day mortality between patients anticoagulated with direct oral anticoagulants (DOACs) and those not anticoagulated at the time of evaluation for an acute hip fracture.

**Design:**

Retrospective chart review Level III Study.

**Setting:**

One university-based hospital in Rochester, NY.

**Patients/Intervention:**

: Patients 65 years and older undergoing operative treatment of a hip fracture over a 5-year period. Chart review identified patients on DOAC therapy at the time of injury as well as an age and sex-matched control group not on anticoagulation.

**Main outcome measurements:**

: Demographics, procedure type, admission/postoperative laboratory work, perioperative metrics, transfusion metrics, discharge course, reoperation, readmission, wound complications, and 30-day mortality were obtained for comparison.

**Results:**

Thirty-six hip fractures anticoagulated on DOACs were compared to 108 controls. The DOAC group had delays to operative treatment (27.6 h, SD 16.3 h, 95% CL [22.0–33.1]) vs the control group (19.8 h, SD 10.5 h, 95% CL [17.7–21.8], *P* = .01). No differences were found in estimated blood loss, procedure time, or change in hemoglobin. Transfusion rates were not significantly different between groups (58.3% DOAC vs 47.2% control, *P* = .25). No difference in reoperation, readmission, wound complication, deep venous thrombosis rates, or 30-day mortality rates were found.

**Conclusion:**

Patients presenting on DOAC therapy at the time of hip fracture have a delay to surgery compared with age and sex-matched controls, but no increase in short term complications or mortality rates. Expedited surgery (within 48 h) appeared to be safe and effective treatment for hip fracture patients on DOAC therapies.

## 1 Introduction

The prevalence of hip fractures continues to rise as the population of the world ages. The total number of hip fractures worldwide is projected to double to 2.6 million by the year 2025 and eventually grow to 4.5 million by the year 2050.^[1]^ Geriatric hip fractures are also associated with significant morbidity and mortality.^[2]^ In an effort to reduce morbidity associated with these injuries, the standard of care has trended toward early operative treatment for these fractures. Numerous studies have shown improved outcomes when patients have a decrease in time to surgical intervention.^[3–7]^

As the elderly population continues to increase, so does the likelihood these patients will be on anticoagulation, as the number of associated comorbidities necessitating anticoagulation is typically higher in this patient population. The subset of patients on newer direct oral anticoagulation (DOAC) has seen a particularly significant increase since Pradaxa (dabigatran) was first approved by the FDA in 2010.^[8]^ Multiple other medications have since been approved for use including: Eliquis (apixaban), Xarelto (rivaroxaban), and Savaysa (edoxaban). These DOAC medications possess a unique mechanism of action that require less testing and can be as efficacious as warfarin for a number of indications.^[9–11]^ The most recent AHA recommendations favor DOAC use over warfarin in patients who require anticoagulation for atrial fibrillation.^[12]^ Studies have shown an overall increase in the number of patients taking DOACs that present to the emergency department as well.^[8,13]^

The increasing incidence of hip fractures coupled with the increasing prescriptions of DOACs is likely to result in a greater frequency of patients who are taking DOACs at the time of hip fracture. However, the influence of DOAC usage on operative treatment, particularly for urgent or emergent procedures, is largely unknown with a number of authors proposing recommendations for management.^[14–16]^ While literature begins to emerge on the safety of DOACs in the setting of urgent procedures, there is minimal literature in the setting of hip fracture. Recent studies focusing on this unique patient population have shown significant delays to surgery and complication rates consistent with the associated delays.^[17–19]^

The purpose of this research was to evaluate if key differences exist in time to surgery, wound complications, length of stay, transfusion rate, and 30-day mortality for patients on DOAC therapy presenting with hip fractures compared to a control group of nonanticoagulated geriatric hip fracture patients. We hypothesized that patients on DOAC therapy would have longer delays to surgery with no increase in transfusion rates, complications, or mortality rates within 30 days of surgery.

## 2 Materials/methods

After institutional review board approval, the billing records from January 2013 to March 2018 were queried for current procedural terminology codes (27268, 27269, 27235, 27236, 27244, 27245, 27248, 27125) that corresponded to femur fracture surgeries. A heterogenous array of codes was chosen to account for potential differences in coding practices among surgeons. Cases were then individually reviewed to ensure they met our inclusion criteria as listed below. The surgeries were performed at a single academic community hospital with a nationally recognized geriatric fracture care center that accepts referrals from the surrounding community and counties of a mid-size northeastern city in New York. Patients who present to this center fall into the general catchment area for the larger academic center. A total of 1001 surgeries were performed by 16 board-certified orthopaedic surgeons, 4 of whom who are fellowship-trained orthopaedic trauma surgeons. All surgeons had residents as assistants in the cases. Inclusion criteria were defined as: Age of 65 years or greater, surgically treated fractures (OTA classification 31-A1-3 and 31-B1-3), DOAC usage at the time of presentation. Exclusion criteria were defined as: Age less than 65 years, concomitant injuries other than a hip fracture, high-energy mechanism, periprosthetic fracture, revision surgery involving removal of previous implants, or pathologic (nonosteoporotic) fractures. A total of 36 fractures in 35 patients on DOAC therapy at the time of hip fracture surgery were identified: 19 were taking Eliquis, 14 Xarelto, 3 Pradaxa.

For the control group, a 3:1 control-to-case ratio was chosen to limit the possibility of a type 2 error given the relatively small study group identified.^[20]^ The control group was identified from the original hip fracture population from the same time frame and subject to the same inclusion/exclusion criteria as the anticoagulated subjects. However, they were not anticoagulated at the time of hip fracture (no DOAC, warfarin, or Plavix). Importantly, patients taking 81 mg of aspirin were allowed for inclusion in the control group given the large prevalence of this medication in the geriatric population. Control patients were chosen consecutively from the original cohort in the same ratio of males to females and with ages plus or minus 2 years for each DOAC patient.

For each case and control, medical records and radiographs were reviewed. Demographic data was collected including biologic sex, age, and the procedure performed. Procedures included long cephalomedullary nail, short cephalomedullary nail, sliding hip screw construct, hemiarthroplasty, and closed reduction percutaneous pinning. Comparison data recorded between the groups included admission and serial inpatient laboratory values such as prothrombin time and international normalized ratio values, hemoglobin, creatinine, and estimated glomerular filtration rate. Age-adjusted Charlson Comorbidity Index and American Society of Anesthesiologist class were calculated or recorded for each group. Comparison peri-operative metrics were reviewed and included time to surgery (TTS), estimated blood loss, length of procedure, use of spinal anesthetic, and need for intensive care unit postoperatively. Hospital course comparisons included changes in hemoglobin, units of packed red blood cells given intra- and postoperatively, and transfusion rate. Discharge data comparisons included length of stay and disposition to home, a skilled nursing facility, hospice, or death during admission. Complication comparisons within 30 days included wound complications, deep venous thrombosis (DVT), reoperation and readmission rates, and 30-day mortality rates. TTS was recorded as the time the patient had an admission order placed in the electronic medical record to the recorded time the subject entered the operating room for surgery. Every patient's record was complete for 30 days postoperatively.

In the postoperative period, protocol at our institution was for all patients who presented on a DOAC to be restarted on their original dosage of DOAC prior to admission on postoperative day 1. The protocol for control patients was to start on a 28-day course of Lovenox that began the morning following surgery. Transfusion threshold for geriatric patients at our institution is a hematocrit < 24% or hemoglobin < 8 g/dL.

Analysis was performed with StatPlus (StatPlus:mac, AnalystSoft Inc.—statistical analysis program for macOS. Version v6. See http://www.analystsoft.com/en/, released 2018). Continuous variables were compared with Mann–Whitney *U* tests and categorical variables were compared with chi-squared testing. The Fisher exact test was used when expected counts were less than 5. Statistical significance was defined as *P* <.05. A *post hoc* power analysis was performed using the mean and standard deviations of the collected data for TTS, demonstrating 80% power with 32 subjects.

## 3 Results

Table 1 shows the demographics, comorbidity scales, and pertinent presenting laboratory values for both the DOAC and control groups.

**Table 1:**
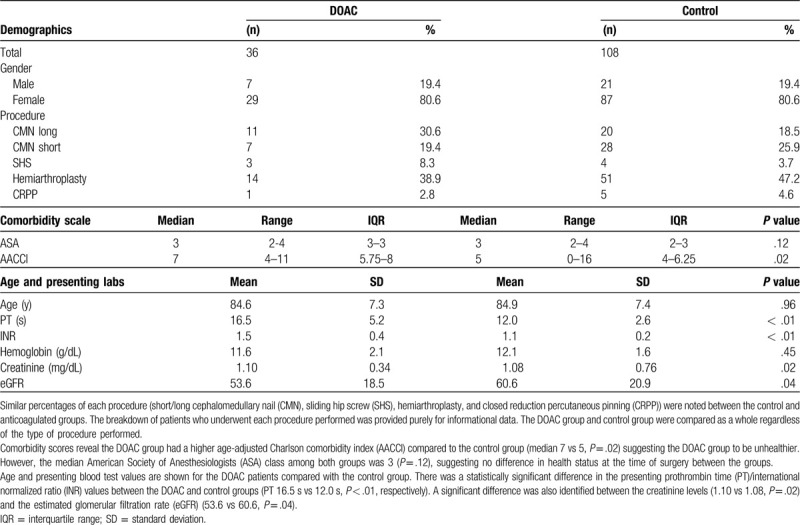
Demographics and preoperative metrics of each group.

Table 2 shows perioperative data including the TTS, estimated blood loss, length of procedure, use of spinal anesthesia, and any patients who required intensive care unit admission postoperatively.

**Table 2:**
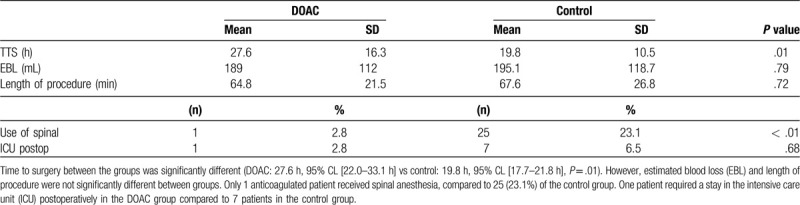
Perioperative metrics.

Table 3 reveals the postoperative outcomes including postoperative changes in hemoglobin (Hgb), transfusion rates, length of stay, readmission/reoperation rates, rate of discharge to a skilled nursing facility, wound complications, and DVT.

**Table 3:**
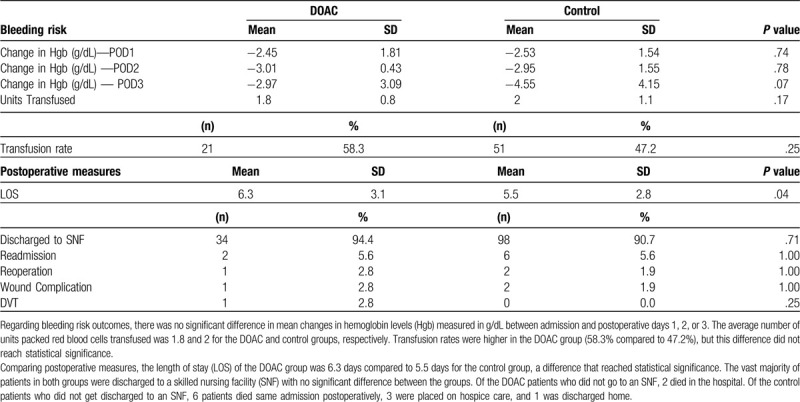
Postoperative metrics.

Regarding postoperative rates of readmission, reoperation, wound complications, and DVT rates refer to Table 3. There were 2 readmissions and 1 patient in the DOAC group can account for the DVT, wound complication, as well as 1 of the readmissions. Thirty-day mortality rates were 8.3% in both the DOAC and the control groups.

## 4 Discussion

The purpose of this study was to evaluate potential differences in the process of care and the postoperative complications for geriatric, low-energy hip fracture patients who present for evaluation on DOACs vs a nonanticoagulated control group. Our data show that no significant differences were observed in complications, blood transfusions rates, or mortality rates in the postoperative short term. Time to surgery and length of stay were the only factors of significant difference between groups, both of which were longer in the DOAC group.

Our study demonstrated that patients on DOACs at the time of hip fracture took longer to receive operative treatment than the control group (average 27.6 h vs 19.8 h). These findings have been shown in numerous other studies in the past, but with longer delays to surgery.^[17–19]^ Schermann et al^[17]^ looked at 1714 patients over the age of 65 undergoing surgery for a hip fracture, with 89 patients on DOAC therapy at the time of presentation, and showed a mean time to surgery of 40.2 hours. Tran et al^[18]^ examined 27 hip fracture patients on DOAC therapy and found a considerably longer mean TTS of 66.9 hours. Reutenberg et al^[19]^ looked at 796 patients with hip fractures, 47 patients on DOAC medication at the time of fracture, and showed a mean TTS of 55.3 hours. Most similar to our study was Franklin et al,^[21]^ who studied 95 geriatric hip fracture patients, with 19 taking DOACs at the time of presentation, and showed a mean TTS of 28.9 hours. In our study, 50% of the DOAC patients underwent operative treatment within 24 hours from admission, and 92% underwent surgery within 48 hours. The quicker average TTS in our DOAC cohort compared with previous studies may be related to a growing familiarity with DOACs in the peri-operative setting as well as the combination of facilities, policies, and specialists that are geared toward expedited surgery in a geriatric fracture care program.

Despite the increased TTS in the DOAC group compared to our controls, the complication rates showed no significant differences in the short term. These findings are mirrored in similar studies previously mentioned. Tran et al reported no increase in rates of DVT, stroke, or death during admission in their cohort of 27 DOAC patients. However, they did not report on wound complications or complications that occurred in follow-up of the patients.^[18]^ Reutenberg et al^[19]^ also reported no significant differences in outcomes or complications in patients who were on DOAC therapy compared to control groups up to 1-year postoperatively. Franklin et al reported on hematoma, persistent serous wound drainage, DVT, readmission, and reoperation in their cohort, showing that DOAC patients had a higher readmission rate when compared with nonanticoagulated controls. The authors attribute this difference to the higher level of comorbidities seen in their DOAC group.^[21]^ The majority of the complications in our DOAC group were attributable to a single patient. The wound complication was a superficial cellulitis successfully treated with a course of oral antibiotics, the deep venous thrombosis was in the setting of this same patient's Factor V Leiden trait, and the readmission/reoperation was for that same patient's hip fracture on the contralateral side from the originally injured hip. The patient was still included in the study because the contralateral hip fracture was greater than 30 days from the primary procedure.

Regarding transfusion rates and 30-day mortality rates, our DOAC group did not show any significant differences compared to the controls. As mentioned before, Schermann et al showed no significant differences in postoperative transfusion rates in 89 DOAC hip fracture patients compared to a nonanticoagulated control group. The authors noted that 1-year mortality was higher in the DOAC group, which they attributed to a sicker patient group on anticoagulation as well as a delayed time to surgery.^[17]^ Reutenberg et al^[19]^ followed their 47 DOAC patient cohort 1 year out and found no differences among in-hospital blood transfusions or 1-year mortality rate compared to a control group. Franklin et al^[21]^ looked at their 19 DOAC patients compared with controls and found postoperative transfusion rates and change in hemoglobin to be similar in both groups. At our institution, the same transfusion threshold guidelines were utilized through the study time period. In a geriatric population, hematocrit of 24% or less, or hemoglobin of 8 g/dL or less prompted transfusion with 1 to 2 units of blood depending on the patient's comorbidity profile. These decisions were usually made in conjunction with the geriatricians that comanage the geriatric patient population. Our study's 30-day mortality rate of 8.3% is similar to previous work done in a larger cohort of 2660 hip fracture patients by Moran et al, which showed a 9% mortality rate. While the study by Franklin et al had a higher readmission rate than our study, their 30-day mortality rate among their 19 DOAC patients was 5.3%.^[4,21]^

While routinely delaying hip fracture surgery beyond 48 hours is likely to increase associated morbidity and mortality, there is an appropriate delay for patients on DOAC therapy. The delay should be based on time of last known dose of the DOAC, the elimination profile of the drug, and the patient's current renal and hepatic function.^[22–24]^ The blood level of the drug of interest may prove some benefit as long as efforts are made to minimize these delays.^[25]^ Leitch and van Vlymen^[26]^ even suggest that a surgical delay of as little as 12 hours from the last known DOAC dose is likely an adequate amount of time to mitigate bleeding risk and an appropriate time frame to minimize the use of reversal agents.

A major limitation with our study was the small sample size of the DOAC group as this provided power only to detect differences in our primary end point of time to surgery, which may underestimate more infrequent events such as bleeding complications or DVT. This limitation can only be overcome with longer, and larger multicenter studies. Our study took place at a single institution with a regionally recognized geriatric fracture care program, which may undercut the generalizability of its findings. As evidenced by the differences in comorbidities index between the groups, the DOAC group was inherently sicker than the control group. Differences between the groups may not be solely attributable to the presence or absence of DOAC therapy, despite our efforts to match the controls with age and sex. Another limitation includes the lack of reliable documentation on the time of last dose of the DOAC provided in our chart reviews. Given that the decision to be “optimized” for the operating room may hinge on time of last dose of a DOAC, this factor could tremendously affect the time to surgery metric. Other limitations of the study include the reporting of blood loss during the procedure and the decision to transfuse the patients. These decisions are subject to reporter variability and are weaknesses inherent to its retrospective nature. We attempted to mitigate to some degree these types of bias by age- and sex-matching our comparison groups.

In conclusion, geriatric patients who presented with a hip fracture in the setting of chronic DOAC therapy had increased time to surgery and length of stay compared to their nonanticoagulated controls but did not significantly differ in their short term postoperative complications, transfusion rates, or 30-day mortality rates. Our study results suggest that no increased short-term risk exists for geriatric patients undergoing expedited (less than 48 h) hip fracture surgery in the setting of DOAC therapy. Further multicenter studies and meta-analyses are required to corroborate our study's findings.
